# Incidence of Mild Traumatic Brain Injury: A Prospective Hospital, Emergency Room and General Practitioner-Based Study

**DOI:** 10.3389/fneur.2019.00638

**Published:** 2019-06-18

**Authors:** Toril Skandsen, Tom Lund Nilsen, Cathrine Einarsen, Ingunn Normann, David McDonagh, Asta Kristine Haberg, Anne Vik

**Affiliations:** ^1^Department of Neuromedicine and Movement Science, Norwegian University of Science and Technology (NTNU), Trondheim, Norway; ^2^Clinic of Physical Medicine and Rehabilitation, St. Olavs University Hospital, Trondheim, Norway; ^3^Department of Public Health and Nursing, Norwegian University of Science and Technology (NTNU), Trondheim, Norway; ^4^Clinic of Anaesthesia and Intensive Care, St. Olavs University Hospital, Trondheim, Norway; ^5^Department of Orthopaedic Surgery, St. Olavs University Hospital, Trondheim, Norway; ^6^Municipal Emergency Department, Trondheim, Norway; ^7^Department of Radiology and Nuclear Medicine, St. Olavs University Hospital, Trondheim, Norway; ^8^Department of Neurosurgery, St. Olavs University Hospital, Trondheim, Norway

**Keywords:** incidence, concussion, mild traumatic brain injury, epidemiological, glasgow coma scale, Norway, emergency room, primary care

## Abstract

**Background:** There are no recent estimates of incidence rates of mild traumatic brain injury (MTBI) from Norway. Moreover, reported incidence rates rarely comprise cases of MTBI evaluated in the primary care setting. In this study, we utilized existing data collected as part of the recruitment to a large, follow-up study of patients with MTBI. We estimated the incidence rate of MTBI, including patients who visited outpatient clinics, in the age group 16–59 years in a Norwegian region.

**Methods:** During 81 weeks in 2014 and 2015, all persons aged 16–59 years, presenting with possible MTBI to the emergency department (ED) at St. Olavs Hospital, Trondheim University Hospital or to the general practitioner (GP)-run Trondheim municipal outpatient ED, were evaluated for a diagnosis of MTBI. Patients were identified by computerized tomography (CT) referrals and patient lists. Patients referred to acute CT from their primary GP with suspicion of MTBI were also recorded. This approach identified 732 patients with MTBI. Age- and sex-specific incidence rates of MTBI were calculated using population figures from the regional catchment area.

**Results:** Overall incidence of MTBI in people between 16 and 59 years was 302 per 100,000 person-years (95% confidence interval 281–324). The incidence rate was highest in the age group 16–20 years, where rates were 835 per 100,000 person-years in males and 726 in females.

**Conclusion:** The overall incidence rate of MTBI was lower than expected from existing estimates. Like other reports, the incidence was highest in the late teens.

## Introduction

Traumatic brain injury (TBI) is a complex injury comprising a spectrum from mild TBI (MTBI) with low risk of persistent disability, to the most severe TBI with devastating brain damage. However, MTBI constitutes 80–90 % of all TBI. It can therefore be appropriate to study characteristics of MTBI, such as incidence, separately. Moreover, a review recently highlighted that the monitoring of the epidemiology of MTBI was incomplete (1). Internationally, the reported incidence rates of mild traumatic brain injury (MTBI) vary extremely, from 100 to 749 cases per 100,000 person-years (2–7). This may reflect a real variation in the burden of traumatic brain injury (TBI), especially in a global context, since injuries are more common in low-income countries (8). However, the variation also results from heterogeneity in study design and data sources (5). Importantly, patients with MTBI who are evaluated outside hospitals, are often not included in incidence estimates (9).

The Nordic countries have been considered safe communities with a decreasing number of TBI (10, 11), but reported rates vary (9, 12). Most of the existing Nordic epidemiological studies on MTBI, however, were conducted decades ago and mostly report incidence rates of hospitalized TBI, either TBI of all severities (9, 11–15) or only MTBI (16). Only a few studies included both hospitalized and non-hospitalized patients (17–19), but none of these dealt exclusively with MTBI.

In the present study, the aim was to estimate the incidence of MTBI in an adult population within a regional Norwegian catchment area. We utilized existing data collected as part of the recruitment to a large, prospective cohort study of patients with MTBI aged 16–59 years (20). Cases were persons who presented with a possible MTBI to the general practitioner (GP) run municipal emergency department (ED) or the ED at a level 1 trauma center, as well as cases referred to computerized tomography (CT) by the patient's primary GP.

## Methods

### Study Period and Setting

Patients aged 16–59 years with MTBI were identified during 81 weeks between April 1st 2014 and December 5th 2015 in two emergency departments (ED): St. Olav's Hospital, Trondheim University Hospital, a Norwegian regional level 1 trauma center and the Trondheim Municipal Emergency clinic (only out-patients), run by the GPs in the area, working shifts. This ED is co-located at the hospital. Their catchment area for MTBI is mostly urban: the city of Trondheim and four neighboring municipal entities with 229,000 residents. The EDs are state run, like most health care in Norway. During normal weekday work hours, patients can contact either their primary GP at one of the 41 GP centers in the catchment area, or one of the EDs. After 3 p.m. on workdays and the whole weekend, patients present to one of the Eds, since the GP centers are closed Mostly, however, patients with mild TBI present directly to the municipal ED at all times of the day. During the study period, indication for CT was assessed with the Scandinavian Guidelines for Head Injury Management from 2000 (21). In these, CT was recommended in patients with either amnesia/ suspected loss of consciousness (LOC) or certain risk factors. Hence, more patients have been referred to CT in Norway than in countries adhering to other, stricter, CT rules.

### MTBI Criteria, and Case Ascertainment

TBI was defined as “an alteration in brain function, or other evidence of brain pathology, caused by an external force” (22), and cases identified with *TBI* were further categorized as *mild* (MTBI) according to the WHO criteria: GCS score 13–15 at presentation, LOC <30 min, and posttraumatic amnesia (PTA) <24 h (23). Patients were identified by daily manual screening of both the patient lists at the EDs and the referrals to head CT, and by daily contact with neurosurgical residents. Notably, also lists of the patients at the municipal ED, who had *not* been referred to CT were prospectively screened. This screening was performed 1–2 times per day by a research assistant who read the notes on all patients who had presented with injuries in the head and neck area or when location was poorly described. Our goal was to identify *all* patients with possible TBI and approach them for case ascertainment. If attempt to contact failed, we used information from the medical records regarding GCS score, amnesia or LOC for MTBI criteria evaluation. Patients who had been referred to head CT from their primary GPs in the catchment area, and met the diagnostic criteria for MTBI were registered, but not approached. For details regarding case ascertainment, see our previous publication (20).

**Figure 1:**
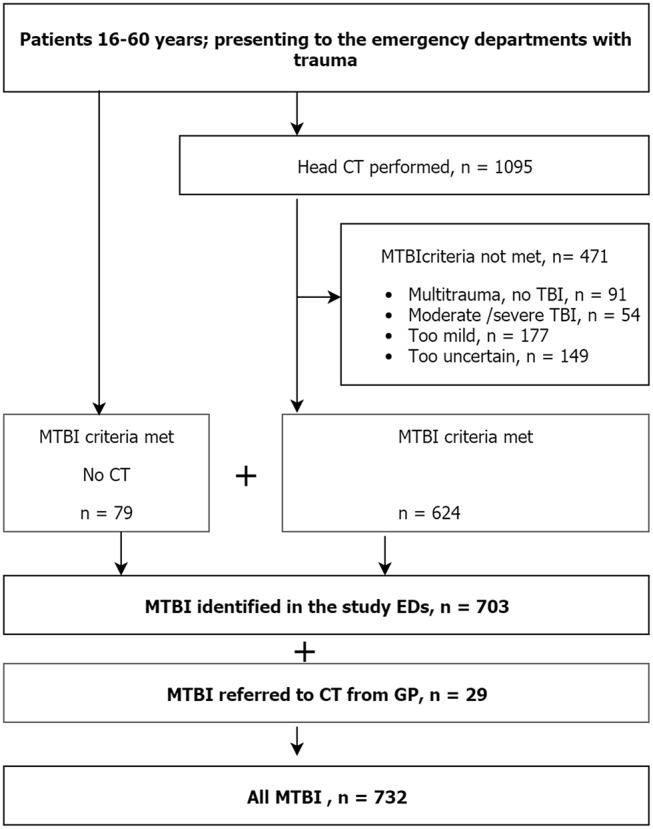
Flow chart of the identification of patients with MTBI. The flow chart demonstrates which figures from the screening procedure that were used for the computation of incidence rates.

During the study period, 1,095 patients were examined with head CT due to trauma, 624 of these were evaluated to have MTBI. Furthermore, 79 patients who had not been examined with head CT, but who had been clinically evaluated to meet the MTBI criteria and enrolled in the original follow-up study were included in the incidence analysis. In addition, 29 patients were retrospectively identified as being directly referred to CT from their primary GP center. Hence, 732 patients were identified with MTBI (Figure 1). As recently published, 517 patients (71%) were treated without hospital admittance and more than 70% had a GCS score of 15. CT showed intracranial lesions in 6%. For details on the clinical and demographic characteristics see (20).

### Incidence Rate Estimation

We estimated age- and sex specific incidence per 100,000 person-years by dividing cases of MTBI during the study period by the population at risk, i.e., total population of catchment area. Cases of MTBI were identified from; (1) all patients who had head CT at the study hospital and who met the diagnostic criteria for MTBI; (2) patients meeting MTBI criteria who eventually consented to participation in the longitudinal study, but who had not been examined with head CT; and (3) patients who had been referred directly to head CT by their primary GPs in the catchment area, and were considered to meet the diagnostic criteria.

The population at risk was obtained from the Norwegian National Registry and Norwegian State Educational Loan Fund, and the total number of people, as well as number of men and women between 16.0 and 59.9 years residing in the catchment area was extracted. This figure included students attending school/college/university in the area, but who had home address elsewhere, and conversely, excluded students with a home address in the catchment area, but who attended school/college/university elsewhere. For the computation of incidence rates, we used the Stata Statistical Software: Release 12. College Station, TX: Stata Corp LP (2011).

## Results

Applying the total of 732 patients who had been identified with MTBI in the catchment area, the overall incidence rate of MTBI in persons aged 16–59 years was 302 per 100,000 person-years (95% CI, 281–324). The incidence rate was 357 (95% CI, 325–391) in males and 242 (95% CI, 216–272) in females. The rate was highest in the age group 16–20 years, where males had a rate of 835 per 100,000 person-years and females a rate of 726 per 100,000 person-years. Table 1 shows the population size, number of MTBI cases, and incidence rates by age groups and sex.

**Table 1:** Mild traumatic brain injury (MTBI) incidence by age and sex.

	**Total population in the study area (*n*)**	**MTBI cases (*n*)**	**Incidence per 100,000 person years (95% CI)**
**Males**			
16–20	7,226	94	835 (682–1022)
21–25	15,536	96	396 (324–484)
26–30	12,358	54	280 (214–366)
31–35	8,801	44	320 (238–431)
36–40	8,219	34	265 (189–371)
41–45	8,435	26	197 (134–290)
46–50	8,122	48	379 (285–503)
51–55	6,886	32	298 (210–421)
56–59	5,198	21	259 (169–397)
Total	80,781	449	357 (325–391)
**Females**			
16–20	6,981	79	726 (582–905)
21–25	14,888	57	245 (189–318)
26–30	10,219	33	207 (147–291)
31–35	8,087	25	198 (134–293)
36–40	7,314	13	114 (66–196)
41–45	7,867	16	130 (79–213)
46–50	7,847	24	196 (131–292)
51–55	6,773	22	208 (137–316)
56–59	5,001	14	179 (106–303)
Total	74,977	283	242 (216–272)

## Discussion

The estimated incidence rates in this study were derived from visits at all medical services available for patients with acute MTBI; the hospital, the GP-run municipal out-patient ED, and the primary GPs in the catchment area. Importantly, 71% were treated without hospital admission. Few other epidemiological studies report such complete coverage. A population-based study from a region in New Zealand also applied case ascertainment at the community level as well as the WHO criteria for MTBI (3). They found an overall incidence rate for MTBI, of 749 per 100,000 person-years across all age groups. Based on their reported data, the incidence of MTBI in the 15–64 year age group can be calculated to 710 per 100,000 person-years, which is substantially higher than in the present study. However, in the New Zealand study, multiple overlapping sources of information about possible cases was used, such as schools and sports organizations, and not only health care providers. Still, the cases identified outside hospitals and GPs in their study constituted only 28%, which cannot explain the large discrepancy in MTBI incidence in the New Zealand study compared to the present study. In line with the estimates from New Zealand, a systematic review on MTBI concluded that the population-based MTBI rate probably is above 600 per 100,000 person-years if accounting for cases not treated at hospitals (2). Hence, in comparison with these figures, the incidence found in the current study using Norwegian data was low.

In contrast, the incidence rate found in the present study corresponds with an overall incidence rate of 354 per 100,000 person-years found in a population based Swedish study of all TBI, where the proportion of MTBI was 97.5% (18). The Swedish study also covered both the catchment hospital and a GP-run ED and assumed to capture all cases of TBI seeking medical evaluation. In that study, only 36% had a CT, while 88% were treated as in-patients, possibly reflecting that the Scandinavian guidelines for TBI from 2000 had not been fully implemented (24). The only Norwegian study covering all medically evaluated head injuries in a defined area, is a retrospective study from Northern Norway in 1993. They found an incidence rate of TBI of all severities of 229 per 100,000 person-years and a rate of hospital admittance of 74% (19) Possibly, the lower estimated incidence in that study, compared to the incidence in the present study, reflects that also in Norway, awareness and recognition of mild TBI has been increasing during the last years, like in the US (25).

In line with most previous studies (18, 19, 26), we found that adolescents in the age group of 16–20 years had much higher incidence rate of MTBI than other ages. It may well be that they have a more active, and possibly more careless, lifestyle involving a higher risk of trauma. Moreover, they typically live with their parents, and may be brought to medical evaluation for a head injury more often.

We assume, however, that the incidence of MTBI was underestimated in the present study since there will always be missed cases of MTBI in a screening process. First, our study procedures did not capture patients seen only by their primary GP unless they were referred to CT, and results must be interpreted with some caution. While it is most common that patients with acute MTBI present directly to the municipal ED, patients who present *more than 24 h* after injury, are more likely to present to their GP, and may not be examined with CT is the risk of intracranial bleeding is over.

Second, we did not register patients not examined with CT, yet meeting criteria for MTBI, unless they were enrolled in the follow-up study. In the recruitment to the follow-up study, we experienced that around 50% of eligible patients eventually got enrolled (20). Hence, the group of 79 consenting patients without CT, should at least be twice as large. If we had calculated with that, the total incidence would, however, only increase to around 330 per 100,000. Seemingly, a large proportion of the patients with MTBI were examined with CT according to our results. We believe that this result reflects the low threshold for CT in the EDs, also shown in a previous publication from our setting (27). Actually, the Nordic countries, and Norway in particular, have been recognized for an increasing use of CT (28). Moreover, only 6% had intracranial findings on CT, and many were examined with CT despite not meeting criteria for MTBI, but rather for minimal head injury, as shown in Figure 1. Third, some patients could not be reached for case assignment, and could be missed for inclusion if clinical signs of MTBI were not clearly described in the record, a recognized source of error in epidemiological studies (29). Fourth, some of the patients evaluated to have “uncertain MTBI” (Figure 1) should possibly have been included. Finally, it should also be mentioned that patients presenting to the EDs with MTBI, but who resided outside the catchment area, were not excluded, likely counterbalanced by the residents in the area who may have sustained MTBI outside the catchment area.

Since there is a large university in the catchment area, there were many students in the population at risk, and the average level of education in the population may be somewhat higher than in Norway as a whole. The incidence of MTBI might therefore not be the same in more rural parts of the country.

Taken together, MTBI was estimated to be less frequent in this Norwegian area than in many other high-income countries, especially among persons older than 20 years. A similar trend has also been shown in previous Norwegian epidemiological studies of all hospitalized (14) and all severe TBI (30). Reasons for the lower incidence might be that people tend to follow security/safety regulations, at home, at work and in the traffic. The true incidence of MTBI, however, will remain unknown, since many patients with MTBI consider it unnecessary to seek medical treatment (31, 32).

In summary, our data indicate that the incidence of medically evaluated MTBI in Norway is lower than 600 per 100,000 person-years anticipated in a recent review, but likely higher than 302 per 100,000 person-years as computed here due to inherent difficulties identifying all MTBI cases as discussed above.

## Ethics Statement

The Regional committee for research ethics approved the study (approval number 2013/754). According to this approval, patients in the follow-up study gave written consent, while consent was not required for use of the information obtained via the screening and case ascertainment procedures.

## Author Contributions

TS, IN, DM, AH, and AV designed the study. TS and TN performed the analyses. TS, IN, and CE collected the data. TS drafted the manuscript. All authors critically reviewed the manuscript. All authors read and approved the last version of this manuscript.

### Conflict of Interest Statement

The authors declare that the research was conducted in the absence of any commercial or financial relationships that could be construed as a potential conflict of interest.
